# Cognitive Compensation Depends on Frontoparietal Network Structure–Function Coupling in Patients With White Matter Hyperintensity

**DOI:** 10.1002/brb3.70871

**Published:** 2025-09-10

**Authors:** Xiao Zhu, Yifei Li, Ying Zhou, Yaode He, Haiwei Huang, Huihong Ke, Jianzhong Sun, Min Lou

**Affiliations:** ^1^ Department of Neurology the Second Affiliated Hospital of Zhejiang University, School of Medicine Hangzhou China; ^2^ Department of Radiology the Second Affiliated Hospital of Zhejiang University, School of Medicine Hangzhou China; ^3^ State Key Laboratory of Transvascular Implantation Devices Hangzhou China

**Keywords:** cognition, compensation, diffusion tensor imaging, functional MRI, white matter hyperintensity

## Abstract

**Background and Purpose:**

White matter hyperintensity (WMH) impairs cognitive function but is not evident in the early stage, raising the need to explore the underlying mechanism. We aimed to investigate the potential role of network structure–function coupling (SC–FC coupling) in cognitive performance of WMH patients.

**Methods:**

A total of 617 participants with WMH (mean age = 61 [SD = 8]; 287 females [46.5%]) were retrospectively included from the Cognitive Impairment, Retinopathy, and Cerebrovascular Lesions in the Elderly (CIRCLE) study who underwent multimodal MRI and comprehensive cognitive assessments. Severe WMH was defined as periventricular WMH with a Fazekas score of 3 and/or deep WMH with Fazekas score ≥ 2; otherwise it was defined as mild WMH. The network SC‐FC coupling was derived from diffusion tensor imaging and functional MRI.

**Results:**

Across networks, the frontoparietal network exhibited the lowest SC–FC coupling, while the somatomotor network was the highest. Within the mild WMH subgroup, only frontoparietal network SC–FC coupling was positively correlated with WMH volume (*β* = 0.136, *p* = 0.014), a pattern not observed in the whole cohort and the severe WMH subgroup (all *p* > 0.05). Furthermore, in the mild WMH subgroup, frontoparietal network SC–FC coupling was also positively correlated with cognitive performance on the digit span forward task, both in cross‐sectional (*β* = 0.110, *p* = 0.023) and longitudinal analyses (*β* = 0.245, *p* = 0.038).

**Conclusions:**

Overall, the frontoparietal network SC–FC coupling may contribute to cognitive compensation during the mild stage of WMH and could be a target for interventions aimed at preserving cognitive abilities.

## Graphical abstract

By integrating functional MRI and DTI, our study found that in patients with the presence of mild white matter hyperintensity (WMH), the frontoparietal network structure–function coupling (SC‐FC coupling) was positively correlated with WMH volume and cognitive function, suggesting its role in cognitive compensation.

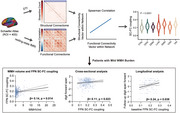

## 1 Introduction

White matter hyperintensity (WMH), characterized by hyperintense signals in T2‐weighted magnetic resonance imaging (MRI) sequences, is one of the imaging markers of cerebral small vessel disease (CSVD) (Wardlaw et al. 2013). WMH has been associated with a higher risk of developing cognitive impairment, particularly in executive function (Alber et al. 2019). Even in patients with mild WMH, around 42% report memory issues (Inzitari et al. 2009). However, the relationship between WMH and cognition in mild cases is less recognized (Zeng et al. 2020), suggesting a need to explore additional neuroimaging mechanisms supporting cognitive discrepancy. Focusing on the mechanism in the early stage provides a possible intervention target to mitigate the WMH progression, which is aligned with the primary goal of clinical trials for CSVD treatments or interventions nowadays (Hamilton et al. 2021).

Though WMH is defined as a structural lesion, it can also disrupt the functional architecture of the brain (Tuladhar et al. 2020). As discovered, structural or functional network disturbance both contributed to cognitive function among patients with WMH. Structural network topology is correlated with information processing speed in CSVD (Wiseman et al. 2018). Meanwhile, the connectivity within functional networks, such as the ventral attention and visual networks, is critical for maintaining cognitive performance in WMH patients (Zhu et al. 2023). However, the existing research mostly used either functional or structural connectivity alone, merely providing insights into the interaction between structural integrity and functional dynamics.

To fill this gap, the concept of structure–function coupling (SC–FC coupling) has been proposed (Hagmann et al. 2010). SC–FC coupling is a fundamental feature that reflects the integrity and flexibility of neural signaling (Avena‐Koenigsberger et al. 2017), which is most frequently measured as the correlation between structural and functional connectivity (Gu et al. 2021). Though a decade has passed, it has only recently been quantified at both the regional and network levels across the cortex (Baum et al. 2020; Sarwar et al. 2021). Physiologically, SC–FC coupling remodels to support functional specialization and cognitive development in youth (Baum et al. 2020). In WMH patients, only SC–FC coupling between the posterior cingulate cortex and medial prefrontal cortex could predict cognitive decline, rather than isolated measures of structural or functional connectivity (Reijmer et al. 2015), suggesting SC–FC coupling as a more accurate and sensitive marker (Fotiadis et al. 2024). Though informative, the possible region‐specific role of SC–FC coupling across the whole cortex has been largely neglected. In a data‐driven study, Du et al. (2025) demonstrated that the structural decoupling index, a novel method for SC–FC coupling calculation, was associated with the brain's hierarchical organization in WMH patients. However, whether network‐level SC–FC coupling detects or compensates for cognitive function in the early stage of WMH remains undiscovered.

In this study, we conducted a retrospective analysis of WMH patients who underwent multimodal MRI from the Cognitive Impairment, Retinopathy, and Cerebrovascular Lesions in the Elderly (CIRCLE) study. Our aim was to investigate the interplay among WMH, network SC–FC coupling, and cognitive function, with a particular focus on patients with mild WMH. This study endeavors to offer novel neuroimaging insights into the substrates of cognitive performance in WMH patients, which may provide a possible target for early intervention.

## 2 Material and Methods

### 2.1 Participant Enrollment

We retrospectively reviewed the data of consecutive patients recruited in the CIRCLE study (ClinicalTrials.gov ID: NCT03542734) between December 2019 and November 2023. The CIRCLE study is a single‐center prospective observational study that enrolls adults (age > 40 years) with sporadic CSVD and without a clinical diagnosis of dementia (neuropsychological, neuroinflammatory, and neurodegenerative diseases) and stroke (both cerebral infarction and hemorrhage), who will undergo assessments on demographics (age, sex, years of education), risk factors (hypertension, hyperlipidemia, diabetes, smoking, and drinking), neuropsychological tests, and multimodal MRI scans. CSVD diagnosis was based on MRI and included the presence of at least one of the following imaging features: recent small subcortical infarcts, lacunes, WMH, enlarged perivascular spaces, microbleeds, or brain atrophy (Wardlaw et al. 2013). Further inclusion criteria of this study included: (1) patients with complete multimodal MRI scans and neuropsychological tests, (2) WMH Fazekas scores > 0 (“WMH Fazekas Scores” in Supporting Information) based on T2 Fluid Attenuated Inversion Recovery (T2 FLAIR). Exclusion criteria were as follows: (1) patients with large head motion during functional MRI scan (i.e., mean power framewise displacement > 0.5 or one of the six head motion parameters > 3 mm/degree); (2) patients with failure of WMH volume calculation due to normalization. In follow‐up analysis, only the participants who completed 2‐year follow‐up MRI and neuropsychological assessments were included.

### 2.2 Neuropsychological Assessment

Physician examination and face‐to‐face interview were administered by trained physicians at baseline. We audiotaped all outcome interviews with permission, and the tapes were randomly selected for review by the supervisor for quality assurance. In the present study, cognitive function was evaluated using the National Institute of Neurological Disorders and Stroke and Canadian Stroke Network (NINDS‐CSN) battery (Chen et al. 2015), Mini‐Mental State Examination (MMSE) (Folstein et al. 1975), and Montreal Cognitive Assessment (MoCA) (Nasreddine et al. 2005). The NINDS‐CSN battery was adapted and validated for the Chinese population (Cronbach's alpha = 0.87, test–retest reliability of subscales *r* = 0.36–0.87) (Chen et al. 2015; Chew et al. 2020). All the neuropsychological assessments were administered by a trained research team to all participants. Detailed tests of the NINDS‐CSN battery, MMSE, and MoCA were in the “Neuropsychological Assessment” of Supporting Information. All individual test scores of the NINDS‐CSN battery were transformed to standardized *z*‐scores. The scores of MMSE and MoCA could represent general cognitive function.

### 2.3 Image Acquisition

All enrolled patients underwent MRI by a 3.0T magnetic resonance scanner using an eight‐channel brain phased array coil. The parameters of the MRI sequence were listed in the “Image Acquisition” of the Supporting Information.

### 2.4 White Matter Hyperintensity

WMH was defined as subcortical hyperintensities without cavitation on T2 FLAIR based on the recommendations of Standards for Reporting Vascular Changes on Neuroimaging (Duering et al. 2023). The criteria of Fazekas scores for periventricular WMH (PWMH) and deep WMH (DWMH) were in the “WMH Fazekas Scores” in Supporting Information. Severe WMH was defined as a PWMH score of 3 or a DWMH score ≥ 2; otherwise, it was defined as mild WMH (Song et al. 2021).

WMH were segmented on T2 FLAIR images using the Lesion Segmentation Tool of Statistical Parametric Mapping launched on MATLAB R2020a (MathWorks, Inc.). First, probabilistic WMH lesion maps were derived from segmentation. Second, the probability threshold was set as 0.5 to convert the probabilistic WMH lesion maps into binary WMH lesion maps. Third, binary WMH lesion maps were manually corrected by X.Z. (MD, 3 years of experience). Fourth, binary WMH lesion maps were co‐registered with T1 using FLIRT, and finally normalized to MNI‐152 space, where WMH volume was extracted. The total volume of WMH was calculated using the labelled voxels multiplied by voxel dimensions.

### 2.5 Construction of Structural Network Connectome

Diffusion Tensor Imaging was processed with the FMRIB Software Library (FSL) and the Pipeline for Analyzing Brain Diffusion Images Toolkit (Cui et al. 2013) as described before (Sun et al. 2022). The edge of the direct white matter structural connectivity was defined using probabilistic tractography. Briefly, the preprocessing procedures included: (1) converting DICOM files into NIFTI images, (2) estimating the brain mask and cropping raw images, (3) correcting for the eddy‐current effect, (4) averaging multiple acquisitions, and (5) calculating diffusion tensor metrics. Then, preprocessed b0 data and crossing fiber modeled diffusion data (called BedpostX data) were obtained.

The brain node was delineated based on the 400‐parcel Schaefer (Schaefer400) (Schaefer et al. 2018) atlas and selected as a seed region. Probabilistic tractography was performed by sampling 5000 streamline fibers for each voxel within the designated seed region. The connectivity probability from the source region to the target region was defined by the number of streamlines passing through the target region divided by the total number of streamlines sampled from the source region. To mitigate underestimations of long‐distance connections within the whole‐brain network, we implemented the distance correction using the –pd option in FSL ProbtrackX tools. The tractography procedure was repeated for all pairs of brain regions to obtain a whole‐brain weighted connectivity matrix for each participant. The weighted connectivity (wij) was computed as the probability of connectivity from seed region i to given region j. Within‐network structural connectivity was defined as the mean wij between each pair of regions of interest (ROIs) within each network.

### 2.6 Construction of Functional Network Connectome

Resting‐state functional MRI and T1 were preprocessed by Data Processing and Analysis of Brain Imaging (Yan et al. 2016). Preprocessing steps for T1 included: (1) converting the DICOM format to NIFTI format, (2) skull stripping, and (3) gray matter, white matter, and cerebrospinal fluid segmentation by Diffeomorphic Anatomical Registration Through Exponentiated Lie Algebra (DARTEL). Preprocessing steps for functional MRI included: (1) convert the DICOM format to NIFTI format; (2) slice timing (removing the first 10 volumes); (3) realignment (to the middle volume), in which 47 participants with mean Power framewise displacement > 0.5 or one of the six head motion parameters > 3 mm/degree were excluded; (4) coregistration with individual T1 image; (5) nuisance regression (i.e., Friston‐24 head motion parameters, white matter signal and cerebrospinal fluid signal which were extracted from individual T1 segmentation); (6) normalization (by DARTEL to Montreal Neurological Institute space into 3 mm × 3 mm × 3 mm voxels), (7) smoothing (with a Gaussian kernel of 6 mm × 6 mm × 6 mm), and (8) band‐pass filtering (0.01–0.1 Hz).

For each subject, the mean resting‐state functional MRI time series of each ROI was extracted and correlated with every other ROI's time course, generating an ROI‐to‐ROI Pearson correlation matrix (*r*‐matrix) based on the Schaefer400 atlas. To increase the normality of the data, the *r*‐matrix was converted into a *z*‐matrix by using Fisher's *z*‐transformation. The *z*‐score is defined as functional connectivity. Within‐network functional connectivity was calculated as the mean ROI‐to‐ROI *z* value between each ROI for each network.

### 2.7 Calculation of Network SC–FC Coupling

The workflow for quantifying network SC–FC coupling is depicted in Figure S1. We used the Schaefer400 parcellation scheme, which integrates local gradient and global similarity and is proven more homogenous than other parcellations (Schaefer et al. 2018). It divides the cortex into seven functional networks according to the resting‐state functional MRI, that is, the frontoparietal network (FPN), the dorsal attention network (DAN), the default mode network (DMN), the limbic network (LMN), the somatomotor network (SMN), the ventral attention network (VAN), and the visual network (VIN).

For each region, the nonzero components of both structural and functional connectivity matrices were extracted and calculated for Spearman correlation, defined as ROI‐wise SC–FC coupling. Network SC–FC coupling was defined as the mean of ROI‐wise SC–FC coupling across all regions in the network. This approach is less sensitive to the effect of outliers and is appropriate when the normal distribution in data cannot be assumed (Gu et al. 2021).

### 2.8 Statistical Analysis

Normality of all the characteristics was checked by the Kolmogorov–Smirnov test and histogram inspection. In the comparison of characteristics between mild and severe WMH subgroups, independent two‐sample *t*‐tests were used for continuous variables and chi‐square tests for binary variables. The association between WMH volume and cognitive performance was tested using linear regression when adjusting for age, sex, and education. To compare network SC–FC coupling in the whole cohort, one‐way repeated ANOVA and post hoc analyses were used. In the comparison of network SC–FC coupling between mild and severe WMH subgroups, linear regression models were used to control for age and sex (group was set as the independent variable). In association of SC–FC coupling, within‐network structural and functional connectivity with WMH volume, Pearson correlation and linear regression analysis (controlled for age and sex) were used. The association between network SC–FC coupling and WMH volume, which survived adjustment for age and sex, was further analyzed by controlling for education and risk factors in a linear regression model. To investigate the association of SC–FC coupling with cognitive tests, baseline and follow‐up cognitive test scores were used as dependent variables for linear regression analysis in cross‐sectional and longitudinal analyses, respectively. Covariates were age, sex, education, and WMH volume for cross‐sectional analysis, and added follow‐up duration and baseline cognitive test scores for longitudinal analysis. Receiver operating characteristic (ROC) curve analysis was used to discriminate patients with follow‐up digit forward span performance in the upper quartile from the lower quartile. We applied the Benjamini–Hochberg false discovery rate (FDR) correction at a significance level of 0.05 to correct multiple comparisons in each group (i.e., the whole cohort, the mild WMH subgroup, and the severe WMH subgroup), respectively. Statistical analyses were performed in SPSS packages (IBM, Chicago, version 22.0 for Windows) and MATLAB R2020a (MathWorks, Inc.). Statistical significance was accepted at the 0.05 level (two‐tailed).

## 3 Results

### 3.1 Participant Characteristics

As shown in Figure S2, 617 participants were finally included in the cross‐sectional analysis (mean age = 61 [SD = 8]; 287 females [46.5%]). Mean WMH volume was 3.78 [SD = 8.00] mL. There were 374 (60.6%) participants with mild WMH, while 243 (39.4%) had severe WMH. Mean power framewise displacement did not show a significant difference between mild (mean = 0.20 [SD = 0.09] mm) and severe WMH subgroups (mean = 0.21 [SD = 0.09] mm) (*t* = −0.77, *p* = 0.44). Table 1 presents detailed vascular risk factors and neuropsychological tests for overall participants as well as for mild and severe WMH subgroups and compares these variables between mild and severe WMH subgroups. Among those in the cross‐sectional analysis, 123 participants were qualified for follow‐up analysis (mean age = 60 [SD = 7]; 43 females [35.0%]). Participants in cross‐sectional analysis had a higher level of education and a greater percentage of females compared to those in longitudinal analysis (Table S1).

**TABLE 1:** Population characteristics.

Characteristics	All (*n* = 617)	Mild WMH (*n* = 374)	Severe WMH (*n* = 243)	*p*
Age (SD)	61 (7)	59 (7)	63 (7)	**< 0.001**
Sex, female (%)	287 (46.5%)	161 (43.0%)	126 (51.9%)	**0.039**
Education [IQR]	9 [6,12]	9 [6,12]	9 [6,12]	**0.043**
WMHV/mL (SD)	3.78 (8.00)	1.11 (1.74)	7.89 (11.42)	**< 0.001**
Risk factors				
Hypertension (%)	450 (72.9%)	257 (69.5%)	193 (79.8%)	**0.005**
Diabetes (%)	137 (22.2%)	79 (21.4%)	58 (24.9%)	0.32
Hypercholesterolemia (%)	264 (42.8%)	161 (43.3%)	103 (42.6%)	0.87
Smoking (%)	232 (37.6%)	153 (41.1%)	79 (32.8%)	**0.041**
Drinking (%)	272 (44.1%)	176 (47.4%)	96 (39.8%)	0.07
Neuropsychological test (SD)				
MMSE	26.3 (3.3)	26.6 (2.8)	25.8 (3.8)	**0.009**
Orientation	9.5 (1.0)	9.6 (0.8)	9.5 (1.2)	0.15
Repetition	2.9 (0.3)	2.9 (0.3)	2.9 (0.3)	0.34
Attention and calculation	4.4 (1.0)	4.4 (0.9)	4.2 (1.1)	**0.03**
Verbal recall	1.6 (1.1)	1.7 (1.0)	1.5 (1.1)	**0.021**
Language	7.9 (1.5)	7.9 (1.5)	7.7 (1.7)	0.14
MoCA	21.9 (4.7)	22.3 (4.3)	21.3 (5.2)	**0.017**
Visuospatial/executive	3.7 (1.3)	3.7 (1.2)	3.5 (1.4)	0.08
Naming	2.3 (0.8)	2.3 (0.7)	2.2 (0.9)	0.15
Attention	5.3 (1.0)	5.4 (1.0)	5.2 (1.1)	0.07
Language	2.0 (1.0)	2.0 (0.9)	2.0 (1.0)	0.49
Abstraction	1.0 (0.8)	1.1 (0.8)	1.0 (0.8)	0.22
Delayed recall	1.7 (1.6)	1.8 (1.6)	1.6 (1.6)	**0.039**
Orientation	5.8 (0.8)	5.9 (0.5)	5.7 (1.1)	**0.01**
NINDS‐CSN battery				
Working memory				
Digit span forward	11.9 (2.7)	12.1 (2.6)	11.6 (2.9)	**0.023**
Digit span backward	5.4 (2.5)	5.5 (2.4)	5.1 (2.7)	0.08
Executive Function				
Verbal fluency	14.2 (3.8)	14.4 (3.8)	13.8 (3.8)	0.09
TMT‐A	−1.90 (0.21)	−1.88 (0.19)	−1.92 (0.23)	**0.026**
TMT‐B	−2.13 (0.18)	−2.11 (0.17)	−2.17 (0.20)	**0.001**
Language				
MBNT	12.4 (1.9)	12.5 (1.7)	12.2 (2.1)	**0.036**
Visuomotor speed				
SDMT	32.9 (12.9)	34.6 (12.1)	30.2 (13.7)	**< 0.001**
Visuospatial function				
RCFT Copy	29.9 (8.4)	30.7 (7.8)	28.7 (9.2)	**0.007**
Memory				
RCFT immediate recall	14.6 (8.2)	15.8 (8.1)	12.7 (8.0)	**< 0.001**
RCFT delayed recall	13.9 (8.3)	15.0 (8.3)	12.3 (7.9)	**< 0.001**
RCFT recognition	18.4 (2.9)	18.5 (3.0)	18.3 (2.7)	0.53
HVLT immediate recall	17.0 (5.5)	17.3 (5.4)	16.6 (5.6)	0.10
HVLT delayed recall	5.8 (2.9)	6.0 (2.8)	5.5 (2.9)	**0.036**
HVLT recognition	8.9 (2.6)	9.2 (2.3)	8.4 (2.8)	**< 0.001**

*Note*: *t*‐test for continuous variables and chi‐square test for binary variables. Bold values indicate statistical significance at p < 0.05.

Abbreviations: HVLT, Hopkins Verbal Learning Test; IQR, interquartile range; MBNT, Modified Boston Naming Test; MMSE, Mini‐Mental State Examination; MoCA, Montreal Cognitive Assessment; RCFT, Rey Complex Figure Test; SD, standard deviation; SDMT, Symbol Digit Modalities Test; WMHV, white matter hyperintensity volume.

### 3.2 Relationship Between WMH Volume and Cognitive Function

#### 3.2.1 General Cognitive Function

The association of WMH volume with general cognitive function is demonstrated in Figure S3. Among all participants, WMH volume was negatively correlated with MMSE (*β* = −0.109, *p* = 0.006, FDR *p* = 0.012) and MoCA (*β* = −0.070, *p* = 0.050, FDR *p* = 0.050). In the severe WMH subgroup, WMH volume had negative correlation trends with MMSE (*β* = −0.130, *p* = 0.036, FDR *p* = 0.072) and MoCA (*β* = −0.090, *p* = 0.09, FDR *p* = 0.09). However, in the mild WMH subgroup, neither MMSE (*β* = 0.021, *p* = 0.69, FDR *p* = 0.69) nor MoCA (*β* = 0.045, *p* = 0.34, FDR *p* = 0.68) was correlated with WMH volume. All analyses above were corrected for age, sex, and education.

#### 3.2.2 Cognitive Domains

The linear regressions of WMH volume with cognitive domains are shown in Table S2 with adjustment for age, sex, and education. Both in the whole cohort and the severe WMH subgroup, WMH volume was negatively correlated with all cognitive domains (all FDR *p* < 0.05) except for language. However, in the mild WMH subgroup, none of the cognitive domains correlated with WMH volume (all FDR *p* > 0.05).

### 3.3 Characteristics of Network SC–FC Coupling

**FIGURE 1:**
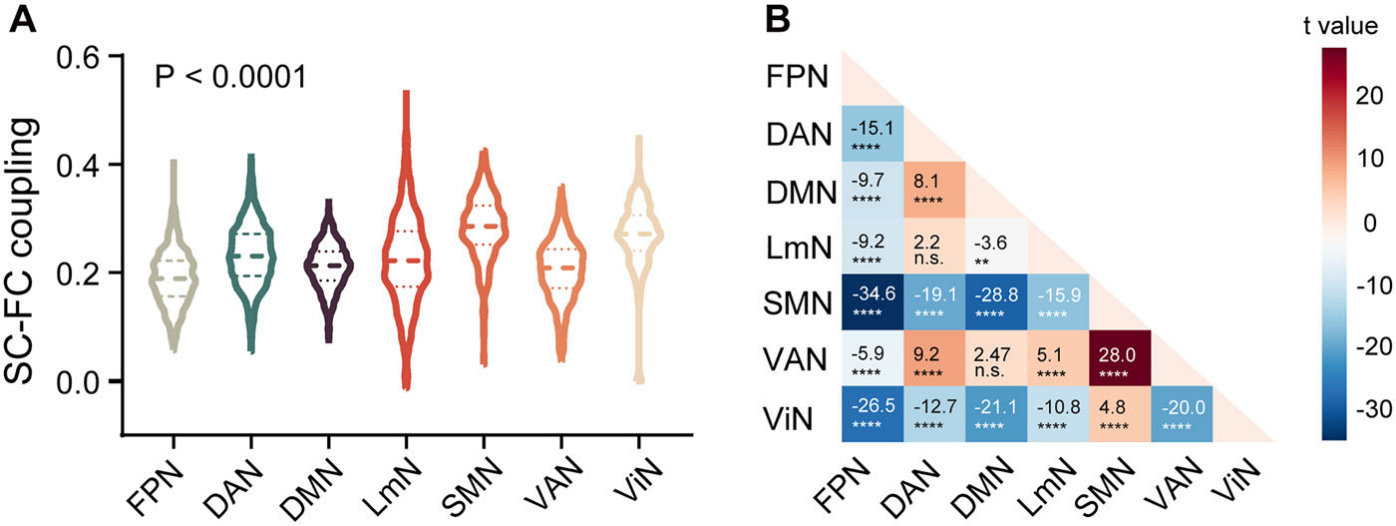
Comparison of structural–functional (SC–FC) coupling among seven networks. (A) Violin plot of SC–FC coupling among networks. *p* indicates the statistical significance of the main effect (i.e., network) for one‐way repeated‐measure ANOVA analysis. SC–FC coupling is of significant difference across seven networks. Violins show the data distribution, and dashed lines represent the maximum, median, and minimum values from top to bottom. (B) Pairwise comparison of structural–functional coupling across networks. Numbers inside each box were *t* values using the Tukey test. Two‐sided *p* values were calculated. DAN, dorsal attention network; DMN, default mode network; FC, functional connectivity; FPN, frontoparietal network; LmN, limbic network; n.s., non‐significance; SC, structural connectivity; SMN, somatomotor network; VAN, ventral attention network; ViN, visual network. **p < 0.01; ****p < 0.0001; n.s. *p* > 0.05.

The network SC–FC coupling is characterized in Figure 1. A one‐way ANOVA test revealed a significant main effect of network on SC–FC coupling (*F*(4.68, 2882) = 246.5, *p* < 0.001). Post hoc analysis found FPN SC–FC coupling was the lowest, while SMN SC–FC coupling was the highest (Figure 1). When controlled for age and sex, only DMN showed higher SC–FC coupling in the severe WMH subgroup than the mild WMH subgroup (*β* = 0.087, *p* = 0.034), whereas other networks didn't show a significant difference between groups.

### 3.4 Relationship Between WMH Volume and Network SC–FC Coupling

**FIGURE 2:**
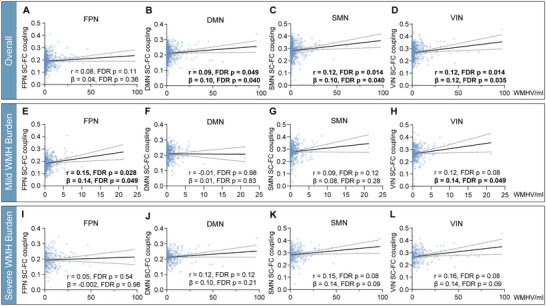
The association of white matter hyperintensity (WMH) volume with network structure–function (SC–FC) coupling. Scatterplot of association between network SC–FC coupling and WMH volume among all participants (A–D), participants with mild WMH burden (E–H), and participants with severe WMH burden (I–L). The linear regression models were corrected for age and sex. Bold indicates significant correlations. The dotted line around the regression line represents the 95% confidence interval for the regression estimate. DMN, default mode network; FDR, false discovery rate; FPN, frontoparietal network; *r*, univariate Pearson correlation coefficient; SMN, somatomotor network; VIN, visual network; β, multivariable linear regression standardized β when adjusting for age and sex.

Pearson correlation and linear regression analyses (adjusting for age and sex) of WMH volume with network SC–FC coupling are shown in Tables S3 and S4, respectively. Among all participants, both analyses revealed positive correlations of WMH volume with DMN, SMN, and VIN SC–FC coupling (all FDR *p* < 0.05) (Figure 2A–D). In the mild WMH subgroup, FPN SC–FC coupling was positively correlated with WMH volume in both analyses (*r* = 0.149, FDR *p* = 0.028; *β* = 0.136, FDR *p* = 0.049), whereas VIN SC–FC coupling was only positively correlated with WMH volume with adjustment for age and sex (*β* = 0.144, FDR *p* = 0.049) (Figure 2E–H). After adjustment for education and risk factors (hypertension, diabetes, hypercholesterolemia, smoking, and drinking), FPN (*β* = 0.122, *p* = 0.033) and VIN (*β* = 0.132, *p* = 0.020) SC–FC coupling remained positively correlated with WMH volume. In contrast, in the severe WMH subgroup, none was significant after FDR correction in Pearson correlation and linear regression analyses when adjusting for age and sex (all FDR *p* > 0.05) (Figure 2I–L).

### 3.5 Relationship Between WMH Volume and Within‐Network Structural and Functional Connectivity

For within‐network structural connectivity, almost all networks were negatively correlated with WMH volume among all participants as well as in subgroups when adjusting for age and sex (all FDR *p* < 0.05, Table S5). However, LMN was only correlated with WMH volume in the mild WMH subgroup, while SMN didn't correlate with WMH volume in the mild WMH subgroup. For within‐network functional connectivity, all networks were negatively correlated with WMH volume in the mild WMH subgroup (all FDR *p* < 0.05, Table S6), but not among all participants or in the severe WMH subgroup when adjusting for age and sex. In the mild WMH subgroup, while both within‐network structural and functional connectivity were generally correlated with WMH volume, only FPN SC–FC coupling was associated with WMH volume, triggering further investigations on the associations between network SC–FC coupling and cognitive function.

### 3.6 Cross‐Sectional Analysis on the Relationship Between Network SC–FC Coupling and Cognitive Function

#### 3.6.1 General Cognitive Function

**FIGURE 3:**
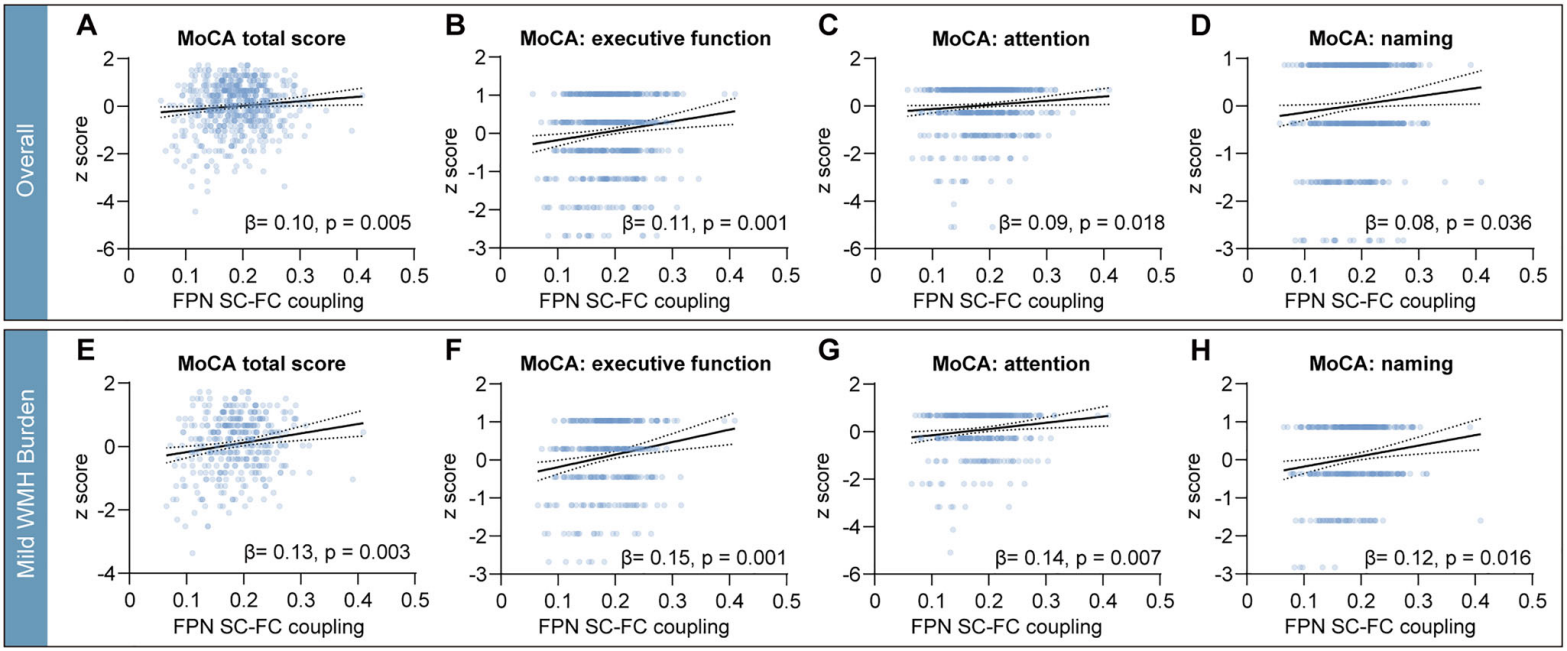
The association of frontoparietal network (FPN) structure–function (SC–FC) coupling with Montreal Cognitive Assessment (MoCA). Scatterplot of association between FPN SC–FC coupling and *z*‐score for MoCA and its subscales among all participants (A–D) and participants with mild WMH burden (E–H). Specifically, the *y*‐axis represents the *z*‐score of the MoCA total score, executive function, attention, and naming from left to right. The linear regression models were corrected for age, sex, education, and white matter hyperintensity (WMH) volume. The dotted line around the regression line represents the 95% confidence interval for the regression estimate. β, multivariable linear regression standardized β.

**FIGURE 4:**
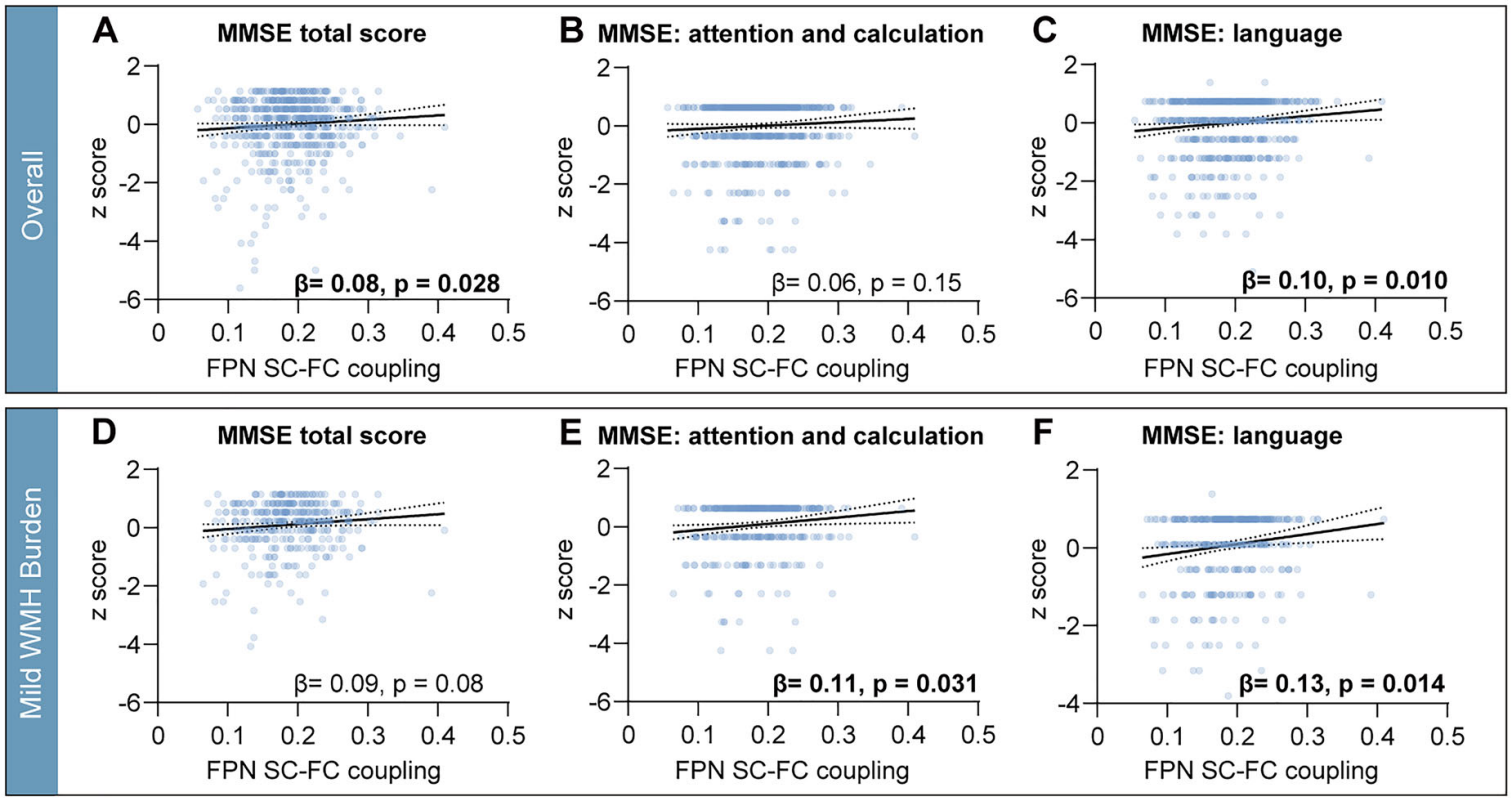
The association of frontoparietal network (FPN) structure–function (SC–FC) coupling with Mini‐Mental State Examination (MMSE). Scatterplot of association between FPN SC–FC coupling and *z*‐score for MoCA and its subscales among all participants (A–C) and participants with mild WMH burden (D–F). Specifically, the *y*‐axis represents the *z*‐score of the MMSE total score, attention, calculation, and language from left to right. The linear regression models were corrected for age, sex, education, and white matter hyperintensity (WMH) volume. The dotted line around the regression line represents the 95% confidence interval for the regression estimate. β, multivariable linear regression standardized β.

As for MoCA, only the mild WMH subgroup showed positive correlations with FPN SC–FC coupling (*r* = 0.167, FDR *p* = 0.007) (Table S7) and survived after controlling for age, sex, education, and WMH volume (*β* = 0.133, *p* = 0.003) (Figure 3E). Except for FPN, no significant correlations were found in other networks. As for MMSE, in the mild WMH subgroup, there was also a positive correlation with FPN SC–FC coupling before FDR correction (*r* = 0.102, *p* = 0.049, FDR *p* = 0.32) (Table S8), and a positive trend after controlling for the above covariates (*β* = 0.087, *p* = 0.08). Except for FPN, only VAN SC–FC coupling was positively correlated with MMSE both among all participants (*r* = 0.114, *p* = 0.005, FDR *p* = 0.035) and in the severe WMH subgroup (*r* = 0.114, *p* < 0.001, FDR *p* < 0.001) (Table S8), and survived after controlling for the above covariates (overall, *β* = 0.092, *p* = 0.013; severe WMH, *β* = 0.167, *p* = 0.004) (Figure 4A,D).

#### 3.6.2 MoCA and MMSE Subscales

Considering the convergent role of FPN SC–FC coupling in relation to WMH volume and cognition, we proceeded to investigate its association with different cognitive domains. Pearson correlations for subscales of MoCA and MMSE are shown in Table S9. As for MoCA subscales, FPN SC–FC coupling was positively correlated with executive function, attention, and naming (all *p* < 0.05) and survived when controlled for age, sex, education, and WMH volume (Figure 3B–D,F–H) both among all participants and in the mild WMH subgroup. However, the severe WMH subgroup didn't show any correlation between FPN SC–FC coupling and MoCA subscales (all FDR *p* > 0.05). As for MMSE subscales, language had a positive trend in correlation with FPN SC–FC coupling (*r* = 0.099, *p* = 0.015, FDR *p* = 0.075), which survived when controlled for covariates above among all participants (*β* = 0.100, *p* = 0.010) (Figure 4C). In the mild WMH subgroup, besides language (*β* = 0.125, *p* = 0.014), attention and calculation (*β* = 0.112, *p* = 0.031) (Figure 4E,F) also showed positive correlation with FPN SC–FC coupling when controlled for covariates above. In the severe WMH subgroup, no correlations were found.

#### 3.6.3 NINDS‐CSN

**FIGURE 5:**
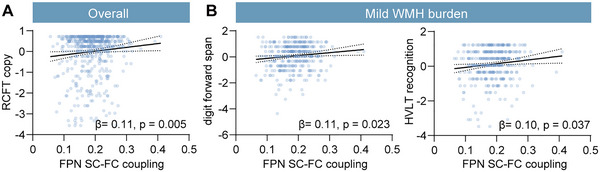
The association of frontoparietal network (FPN) structure–function (SC–FC) coupling with cognitive tests in the NINDS‐CSN battery. (A) Scatterplot of association between FPN SC–FC coupling and the *z*‐score of the Rey Complex Figure Test (RCFT) copy among all participants. (B) Scatterplot of the association between FPN SC–FC coupling and *z*‐score of digit forward span and Hopkins Verbal Learning Test (HVLT) recognition in the mild white matter hyperintensity (WMH) burden subgroup. The linear regression models were corrected for age, sex, education, and WMH volume. The dotted line around the regression line represents the 95% confidence interval for the regression estimate. β, multivariable linear regression standardized β.

As for NINDS‐CSN, FPN SC–FC coupling was positively correlated with RCFT copy among all participants (*r* = 0.090, *p* = 0.025) (Table S10) before FDR correction and survived after correcting for age, sex, education, and WMH volume (*β* = 0.106, *p* = 0.005) (Figure 5A). In the mild WMH subgroup, FPN SC–FC coupling was positively correlated with digit span forward, HVLT recognition, verbal fluency, and MBNT (all *p* < 0.05) (Table S10) before FDR correction. After adjusting for covariates above, only digit span forward (*β* = 0.110, *p* = 0.023) and HVLT recognition (*β* = 0.102, *p* = 0.037) remained positive (Figure 5B). No cognitive tests were correlated with FPN SC–FC coupling in the severe WMH subgroup (all *p* > 0.05) (Table S10).

### 3.7 Longitudinal Analysis on the Relationship Between Network SC–FC Coupling and Cognitive Function

**FIGURE 6:**
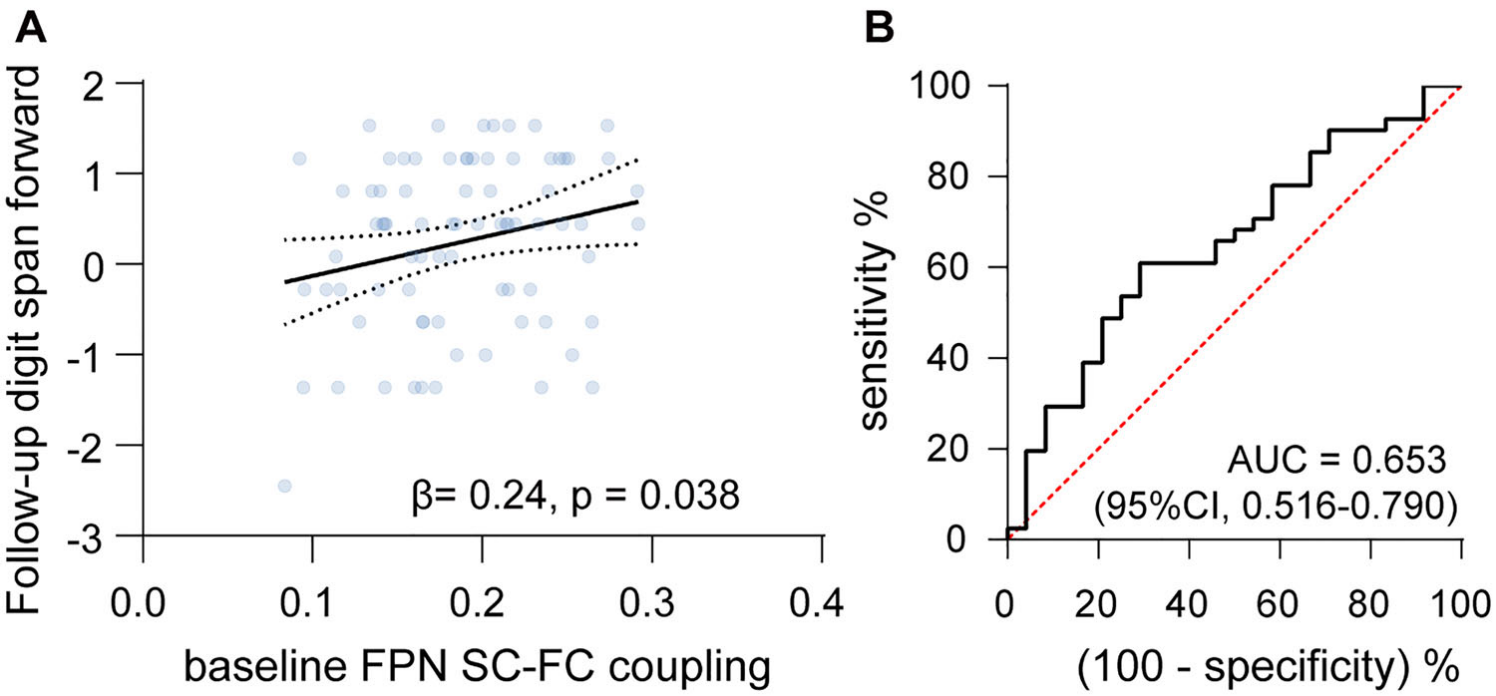
Longitudinal analysis in the mild white matter hyperintensity (WMH) subgroup. (A) Scatterplot of association between baseline frontoparietal network (FPN) structure–function (SC–FC) coupling and *z*‐score of follow‐up digit span forward. (B) Receiver Operating Characteristic (ROC) curve for discriminating the upper quartile of follow‐up digit span forward from the lower quartile when setting baseline FPN SC–FC coupling as the dependent variable. 95% CI, 95% confidence interval; AUC, area under the ROC curve.

In longitudinal analysis, there were no significant correlations of baseline FPN SC–FC coupling with follow‐up MoCA or MMSE total scores and related subscales in the mild WMH subgroup (all *p* > 0.05). However, baseline FPN SC–FC coupling was positively correlated with follow‐up digit span forward when adjusting for age, sex, education, baseline WMHV, baseline digit span forward, and follow‐up duration in the mild WMH subgroup (*β* = 0.245, *p* = 0.038) (Figure 6A). No significant results were found among all participants and in the severe WMH subgroup (all *p* > 0.05).

### 3.8 ROC Analysis

For discrimination based on upper and lower quartiles of follow‐up digit span forward scores, the area under the curve of the ROC analysis was 0.653 (95% CI, 0.516–0.790) when based on baseline FPN SC–FC coupling in the mild WMH subgroup (Figure 6B). To identify individuals at a higher risk for longitudinal decline in the digit span forward test, a cutoff value for FPN SC–FC coupling was established at 0.144, which yielded a high sensitivity of 0.905.

## 4 Discussion

In this large‐scale imaging study, we identified a compensatory role for FPN SC–FC coupling in cognitive function, especially during the early stage of WMH. Interestingly, within the mild WMH subgroup, FPN SC–FC coupling increased as WMH expanded. Furthermore, this enhancement in FPN SC–FC coupling at the mild stage was associated with better performance on the digit span forward task in both cross‐sectional and longitudinal analyses. To identify individuals with mild WMH who were at higher risk of developing cognitive decline during follow‐up, we established a threshold for FPN SC–FC coupling at 0.144, which demonstrated 90% sensitivity. Overall, FPN SC–FC coupling may serve as a promising imaging biomarker for predicting cognitive decline and offer valuable insights for personalized treatment strategies in WMH patients.

Inconsistent with network SC–FC coupling, WMH exerted a dual effect on both within‐network structural and functional connectivity. Specifically, while the function of all networks was disrupted in the early stage of WMH, the structure continued to deteriorate as WMH progressed. The widespread decrease in structural and functional connectivity, however, couldn't specifically explain the cognitive heterogeneity in mild WMH patients. Conversely, only FPN SC–FC coupling increased as WMH progressed in the mild stage, which was also observed by another study (Tay et al. 2023). This finding prompted us to dive into mechanisms underlying the cognitive heterogeneity in the early stage.

Among patients with WMH, the SC–FC coupling across various networks was lowest in the FPN, and highest in SMN. This finding is in line with developmental and aging studies (Griffa et al. 2022; Liu et al. 2023), which both show a gradient of SC–FC coupling that decreases from unimodal to transmodal cortex (Paquola et al. 2019). SC–FC coupling seems to maintain a stable pattern across cortical networks in different populations. Notably, the positive correlation between FPN SC–FC coupling and WMH volume was observed exclusively in the mild WMH subgroup. The trajectory of FPN SC–FC coupling in response to WMH aligns with the “last in, first out” theory, positing that regions supporting higher‐order cognitive functions take longer to mature but are more vulnerable to aging (Fjell et al. 2014). In the early stage, the spatial preference of WMH in the frontal lobes may be the basis underlying the observed increase in FPN SC–FC coupling (Li et al. 2023; Mayer et al. 2021). The biological mechanisms driving changes in SC–FC coupling likely involve intracortical myelination and excitation‐inhibition balance (Fotiadis et al. 2023). Specifically, intracortical myelination provides the structural foundation (Knowles et al. 2022), while the excitation‐inhibition balance reflects the functional characteristic (Fotiadis et al. 2023). From granular to agranular cortex, the reliance of network SC–FC coupling shifts from the excitation‐inhibition balance to intracortical myelination (Fatterpekar et al. 2002). The FPN predominantly situates in the agranular cortex, and its SC–FC coupling relies more heavily on intracortical myelination (Fotiadis et al. 2023), which is interconnected through extracortical white matter connections (Demirtaş et al. 2019; Vazquez‐Rodriguez et al. 2019). Consequently, FPN might be more vulnerable to deep WMH by influencing connected intracortical myelination, which then reorganizes the structural basis and increases SC–FC coupling. In addition to intracortical myelination, WMH could also affect neurovascular coupling (Yang and Webb 2023) and disrupt the balance of excitatory and inhibitory activities, potentially inducing changes in SC–FC coupling. In the late stage, FPN may exceed their extremity in coping with WMH lesions, resulting in a nonsignificant correlation between WMH volume and FPN SC–FC coupling. However, it is important to note that the mechanisms proposed are primarily inferred from imaging studies and have not yet been causally validated. Future research should focus on elucidating the molecular mechanisms underlying the increased FPN SC–FC coupling with WMH progression in the early stage.

Concurrently, the adaptive increase in FPN SC–FC coupling also showed a positive correlation with cognitive function, particularly within the domains of executive function and working memory. We propose two potential mechanisms. One is the compensation. The increased FPN SC–FC coupling may serve as a compensatory response to lesions in maintaining intact cognitive function. It aligns with studies suggesting that increased neural activity, particularly in the frontal region, can counteract age‐related cognitive decline (Cabeza et al. 2002; Grady et al. 2003). The other is the nonselective recruitment. Alternatively, the observed increase in SC–FC coupling simply represents a nonselective recruitment of neural resources, indicating a more generalized activation that may not necessarily correlate with improved performance (Logan et al. 2002). Given the strong association among FPN SC–FC coupling, WMH progression, and cognitive performance, we hypothesize that the FPN is more likely to fulfill a compensatory role. FPN participates in executive function and working memory (Dixon et al. 2018), and its neural activity increases to cope with elevated cognitive burden (Spreng et al. 2010), providing a basis for cognitive compensation. In a developmental cohort, increased FPN SC–FC coupling has been linked to enhanced general intelligence (Feng et al. 2024), highlighting the role of FPN in cognitive maturation. Moreover, in an aging population, FPN functional connectivity has been shown to confer greater resilience against memory impairments associated with early tau pathology (Neitzel et al. 2019), offering preliminary support for the compensatory role of FPN with the presence of mild neuropathology. On the contrary, in the early stage, VIN SC–FC coupling also increased as WMH volume progressed but wasn't correlated with general cognitive function, indicating a higher possibility of nonselective recruitment rather than compensation. Together, the above evidence supports the notion that FPN SC–FC coupling may play a compensatory role in enduring WMH in the early stage.

The SC–FC coupling is of higher predictive accuracy in identifying individuals with cognitive impairment when compared with the isolated use of structural or functional network characteristics (Reijmer et al. 2015), underscoring its promising translational potential in early diagnosis and intervention (Fotiadis et al. 2024). Moreover, SC–FC coupling is recognized as a dynamic and plastic imaging feature (Zamani Esfahlani et al. 2022), suggesting that it may be modifiable through interventions such as transcranial direct current stimulation (Chiang et al. 2022; Eren and Yilmaz 2022). Overall, FPN SC–FC coupling could be utilized as a neuroimaging biomarker to predict cognitive function and a novel intervention target to enhance cognitive function in early WMH patients.

Several limitations of our study need to be considered. First, we only investigated network‐level SC–FC coupling in this study, since cognitive performance relies on intact network function and structure (Yeo et al. 2011). Future work should investigate how regional SC–FC coupling changes in the context of WMH and whether some hub regions coordinate network SC–FC coupling, potentially shedding light on screening biomarkers as well as intervention targets. Second, we used Spearman correlation to calculate SC–FC coupling. While this approach is widely used and provides valuable insights (Fotiadis et al. 2024), several advanced methodologies are arising. The harmonic analysis approach (Preti and Van De Ville 2019) and the modelling approach (Vazquez‐Rodriguez et al. 2019), though of different calculations, both utilize the WMH‐related weighted disconnectome, offering alternative ways to investigate SC–FC coupling at both network and regional levels. Third, the difference of gender and years of education between participants in cross‐sectional and longitudinal analyses, as well as the limited follow‐up rate, which is mainly due to the single‐center design and the suspensions during 2020–2022 due to the COVID‐19 impact, warrants great caution in interpreting results concerning possible bias. Fourth, the distribution of cognitive test scores has shown floor or ceiling effects, limiting its statistical power in correlation analysis. Fifth, the univariate correlation between digit forward span and FPN SC–FC coupling couldn't survive after FDR correction, though it remained significant in multivariate regression and longitudinal analysis. Hence, the interpretation of these results warrants great caution. Sixth, due to the retrospective nature of the study, our study may include participants with a higher level of education and compliance; thus, we could not rule out the possible selection bias, which may have affected the results. Furthermore, the potential impact of unmeasured factors, such as genetic factors or lifestyle factors not included in the analysis, has to be considered in future investigations. Finally, only the Schaefer400 parcellation scheme was used in the present study. Though it is of higher homogeneity and wider use compared to others, our results need to be verified in other parcellation schemes.

Integrating both cross‐sectional and longitudinal cohorts, here we illustrated how SC–FC coupling within neural networks evolves with the presence of WMH and clarified its compensatory role in preserving executive function and working memory, especially during the early stage. Our findings not only underscore the potential of FPN SC–FC coupling as a predictive biomarker for cognitive performance but also suggest it as a novel target for early intervention strategies in WMH patients.

## Author Contributions

**Xiao Zhu**: conceptualization, methodology, data curation, investigation, validation, formal analysis, visualization, project administration, writing – original draft. **Yifei Li**: conceptualization, methodology, data curation, investigation, validation, formal analysis, visualization, project administration, writing – original draft. **Ying Zhou**: writing – review and editing, project administration. **Yaode He**: writing – review and editing, project administration. **Haiwei Huang**: project administration, writing – review and editing. **Huihong Ke**: project administration, writing – review and editing. **Jianzhong Sun**: resources, project administration, writing – review and editing. **Min Lou**: conceptualization, supervision, funding acquisition, resources, writing – review and editing.

## Ethics Statement

All subjects had been given written informed consent prior to the study, and the protocols had been approved by the Human Ethics Committee of the Second Affiliated Hospital of Zhejiang University. All clinical investigations have been conducted according to the principles expressed in the Declaration of Helsinki.

## Conflicts of Interest

The authors declare no conflicts of interest.

## Peer Review

The peer review history for this article is available at https://publons.com/publon/10.1002/brb3.70871.

## Supporting information




**Supplementary Materials**: brb370871‐sup‐0001‐SuppMat.docx

## Data Availability

The datasets generated and analyzed for this study are available from the corresponding author after publication upon reasonable request.
